# Continuity of medication information transfer and continuous medication supply during hospital-to-home transitions - nationwide surveys in hospital and community pharmacies after implementing new legal requirements in Germany

**DOI:** 10.1186/s12913-024-11208-4

**Published:** 2024-08-27

**Authors:** Sophia Klasing, Frank Dörje, Heike Hilgarth, Nadine Metzger, Ina Richling, Hanna M. Seidling

**Affiliations:** 1 Heidelberg University, Medical Faculty Heidelberg/Heidelberg University Hospital, Internal Medicine IX – Department of Clinical Pharmacology and Pharmacoepidemiology, Cooperation Unit Clinical Pharmacy, Im Neuenheimer Feld 410, 69120 Heidelberg, Germany; 2 Joined Discharge Management Project Group of the Federal Association of German Hospital Pharmacists (ADKA) e.V. and the German Pharmaceutical Society (DPhG) e. V., Alt-Moabit 96/Varrentrappstraße 40-42, 10559/60486 Berlin/Frankfurt am Main, Germany; 3 Pharmacy Department, Universitätsklinikum Erlangen and Friedrich-Alexander-Universität Erlangen-Nürnberg, Palmsanlage 3, 91054 Erlangen, Germany; 4 Federal Association of German Hospital Pharmacists (ADKA) e. V./ADKA Academy of Hospital Pharmacy gGmbH, Alt-Moabit 96, 10559 Berlin, Germany; 5 German Pharmaceutical Society (DPhG) e. V, Varrentrappstraße 40-42, 60486 Frankfurt am Main, Germany; 6 Central Pharmacy of the catholic clinics of Märkischer Kreis (Zentralapotheke der Katholischen Kliniken im Märkischen Kreis), Hochstraße 63, 58638 Iserlohn, Germany; 7 Pharmacy, St.-Johannes-Hospital, Johannesstraße 9-13, 44137 Dortmund, Germany

**Keywords:** Continuity of patient care, Hospital-to-home transitions, Drug therapy, Health information exchange

## Abstract

**Background:**

While successful information transfer and seamless medication supply are fundamental to medication safety during hospital-to-home transitions, disruptions are frequently reported. In Germany, new legal requirements came into force in 2017, strengthening medication lists and discharge summaries as preferred means of information transfer. In addition to previous regulations – such as dispensing medication at discharge by hospital pharmacies – hospital physicians were now allowed to issue discharge prescriptions to be supplied by community pharmacies. The aim of this survey study was to gain first nationwide insights into how these requirements are implemented and how they impact the continuity of medication information transfer and continuous medication supply.

**Methods:**

Two nationwide self-administered online surveys of all hospital and community pharmacies across Germany were developed and conducted from April 17th to June 30th, 2023.

**Results:**

Overall, 31.0% (*n* = 111) of all German hospital pharmacies and 4.5% (*n* = 811) of all community pharmacies participated. The majority of those hospital pharmacies reported that patients who were discharged were typically provided with discharge summaries (89.2%), medication lists (59.5%) and if needed, discharge prescriptions (67.6%) and/or required medication (67.6%). About every second community pharmacy (49.0%) indicated that up to half of the recently discharged patients who came to their pharmacy typically presented medication lists. 34.0% of the community pharmacies stated that they typically received a discharge summary from recently discharged patients at least once per week. About three in four community pharmacies (73.3%) indicated that most discharge prescriptions were dispensed in time. However, one-third (31.0%) estimated that half and more of the patients experienced gaps in medication supply. Community pharmacies reported challenges with the legal requirements – such as patients´ poor comprehensibility of medication lists, medication discrepancies, unmet formal requirements of discharge prescriptions, and poor accessibility of hospital staff in case of queries. In comparison, hospital pharmacies named technical issues, time/personnel resources, and deficits in patient knowledge of medication as difficulties.

**Conclusion:**

According to the pharmacies´ perceptions, it can be assumed that discontinuation in medication information transfer and lack of medication supply still occur today during hospital-to-home transitions, despite the new legal requirements. Further research is necessary to supplement these results by the perspectives of other healthcare professionals and patients in order to identify efficient strategies.

**Supplementary Information:**

The online version contains supplementary material available at 10.1186/s12913-024-11208-4.

## Background

Hospital-to-home transitions endanger medication safety – for example, it is reported that every second discharged patient experiences medication errors or medication discrepancies, and one in five suffers from adverse drug events [1]. There are reports that potentially inappropriate medication and/or potential prescribing omissions in patients´ discharge medication jeopardise patient safety (e.g. contributing to readmissions) [2] and that medication-related harm could often be preventable [3].

During hospitalisation, nearly all patients (98.1%) experience at least one and frequently five or more changes in their medication [4]. While patients are closely supervised during hospitalisation, they have to take on a more active role in organising and implementing their drug treatment immediately after discharge [5]. Hence, it is crucial to successfully pass on information on the planned treatment to both patients and outpatient healthcare professionals and to facilitate understanding and access to medication after discharge. This is enforced by the World Health Organization`s Global Patient Safety Challenge *‘Medication without harm’* which declares *‘Medication safety in transitions of care’* as one of the three prioritised aims (others: *‘medication safety in polypharmacy and high-risk situations’*) [6]. However, discontinuities in communication between inpatient and outpatient care settings still occur in routine care [7] and are typically due to poor medication information quality or delayed accessibility of discharge summaries [8–11].

In Germany, > 16 million discharges are reported yearly [12]. To ensure information continuity during hospital-to-home transitions, hospital physicians inform primary care physicians predominantly by means of written discharge summaries. These paper-based or electronic documents are handed over to patients at discharge and/or sent to primary care physicians. The discharge summaries generally contain crucial information – such as reasons for hospitalisation, medical history, course of hospitalisation, inpatient procedures or treatments as well as a list of recommended actions, including medication after discharge [13]. In 2017, new legal requirements (Table 1) came into force. These specified how discharge medications should be documented in discharge summaries and that patients should receive these in addition to written medication lists. The latter also provide information about current medications, dosages and patient-centred advices for administration [13]. To address potential gaps in medication supply, the new legal requirements enabled hospital physicians to issue so-called discharge prescriptions and hand those over to patients at discharge. The prescriptions can then be filled in in any community pharmacy [13]. They differ from prescriptions in primary care in terms of the permitted package sizes to be prescribed (smallest package size only) and validity (three workdays only). Additionally, the physicians who issue the prescription need to be specialists (and no interns) [14]. Discharge prescriptions supplemented the already existing option of providing medications for up to three days by the hospital pharmacy (only before/on weekends or public holidays) [15].

**Table 1:** Changes introduced by the new legal requirements for the discharge management in Germany in 2017

	Before 2017, …	After the new legal requirements came into force in 2017, …
Continuity of medication information transfer	discharge summaries were issued according to hospital or clinic specific standards.	a national standard was set to specify requirements for medication documentation in **discharge summaries** [13].
	medication lists could be handed over to patients but were not mandatory [16].	**medication lists** are to be handed over to all patients with medication at discharge [13].
Continuous medication supply	hospital physicians were not allowed to issue prescriptions for patients who were discharged.	so-called ‘**discharge-prescriptions**’ were introduced to be issued by hospital physicians, handed over to patients who then fill these in in community pharmacies to receive their required medication [13].
	it was possible to dispense medication for up to three days upon discharge before weekends/public holidays [15]	it is still possible to **dispense medication** as before [15].

Even if the new legal requirements do not specify the role of community and hospital pharmacies in the discharge management process, both are often regularly involved in routine care. During hospitalisation, hospital pharmacies may be involved in preparing/supporting issuance of medication lists and discharge summaries [17]. In comparison, community pharmacies are the first point of contact for patients´ after discharge to ensure medication supply. If needed, they may perform medication reviews for patients with polypharmacy which are reimbursed by the health insurance [18, 19].

Five years after the implementation of the legal requirements, it is still unknown (i) whether and how (e.g. using which technologies and methodologies) these requirements are implemented in routine care, (ii) how well they are implemented (e.g. whether there are workarounds or challenges in daily implementation), (iii) which barriers and facilitators exist in implementation, and (iv) what influence these requirements have on the continuity of medication information transfer and continuous medication supply.

The aim of the survey study presented here was therefore to provide initial nationwide insights into current routine care during hospital-to-home transitions from pharmacists´ perspective – e.g. how these requirements are implemented and how they impact the continuity of medication information transfer and continuous medication supply.

## Methods

As part of this survey study, two nationwide, self-administered online surveys of hospital and community pharmacies (one survey for each group) were conducted.

The present evaluation is reported according to the *Consensus-Based Checklist for Reporting of Survey Studies* (CROSS) [20].

### Survey development

Both surveys were conceptualised, developed, piloted, conducted and evaluated by a group of research pharmacists of the Cooperation Unit of Clinical Pharmacy, University Hospital Heidelberg (mainly by SK, HS). This was done in conceptual coordination with pharmacists of the joined discharge management project group of the Federal Association of German Hospital Pharmacists (ADKA) e. V. and the German Pharmaceutical Society (DPhG) e. V. (led by FD, HH, NM, IR). They proposed topics from their working experiences, reviewed the questions and supported the testing and conduction of the surveys. ADKA consists of roughly 2,500 voluntary members [21] – covering about 85% of all hospital pharmacists [22]. It represents the hospital pharmacists` interests vis-à-vis other national and international associations as well as the public and politicians. ADKA also promotes research and continuing education tailored for hospital pharmacists [23]. DPhG is a research association of pharmacists and consists of roughly 10,000 voluntary members [24] which are German pharmacists, students of pharmacy, pharmaceutical institutes and university departments. In comparison to all German pharmacists, it covers about 14% [22]. It facilitates research interests and continuing education in all fields of pharmacy (e.g. clinical pharmacy, community pharmacy, industrial pharmacy) [25].

The surveys´ questions were developed according to the principles of Faulbaum et al. [26] and were examined for comprehensibility, clarity, and unambiguity according to the quality assessment criteria of Faulbaum et al. [26] (conducted independently by SK and a pharmacist in training of the Cooperation Unit of Clinical Pharmacy, University Hospital Heidelberg). Both pre-final online surveys were piloted in individual interviews with seven community pharmacists and seven hospital pharmacists (via videophone; audio-recorded). Therefore, think-aloud and cognitive interviewing techniques were used to ensure the comprehensibility and feasibility of the survey procedure for the respective targeted groups [26]. As needed, the questions were iteratively adjusted. In case of unambiguity or relevant changes in content and wording, these were discussed and consented with the working group.

### Structure of the surveys

In terms of content, both surveys were divided into six different sections (Table 2; see Additional file 1 for non-validated translations of these surveys). This paper focuses on the results of Sects. 1, 2, 4, and 5 that refer to the implementation of medication lists, discharge summaries, discharge prescriptions, dispensing drugs upon discharge) [13] as well as their barriers and facilitators to implementation.

Most of the questions were closed-ended questions with single or multiple-choice answers and contained only a few text input options for further, non-predefined responses. The questions could be obligatory and/or conditional, with conditional questions only being asked if they applied according to the response patterns of previous question(s). For example, only hospital pharmacies that stated that they were actively involved in issuing and handing out medication lists were asked about their experiences and difficulties in routine care. In addition, if respondents had chosen the answer ‘no difficulties’ or ‘unable to assess’, further answer options of the respective questions were hidden to avoid implausible response patterns.

**Table 2:** The structure of the surveys

		Addressee								
		Hospital pharmacies					Community pharmacies			
Theme of section		Number of questions per section								
		*oq*	*q*	*cq*	*t*		*oq*	*q*	*cq*	*t*
1.	General and sociodemographic questions	4	4	0	8		5	6	1	12
2.	General questions about the new legal requirements (e.g. current barriers to implementation)	3	5	3	11		0	1	2	3
3.	Regular involvement of pharmaceutical staff in clinical processes, including discharge management	1	0	0	1		na	na	na	na
4.	Medication information transfer									
	Medication lists	0	0	7	7		1	0	3	3
	Patient consultation	0	0	7	7		0	0	3	3
	Discharge summaries	0	0	6	6		1	0	2	3
5.	Continuous medication supply									
	Discharge prescriptions	0	0	7	7		2	0	3	5
	Dispensing medication upon discharge	0	0	8	8		na	na	na	na
6.	Potential for improvement of discharge processes	0	3	0	3					2
total					58					31

oq = obligatory question which had to be answered by every respondent; q = question which could be answered by every respondent; cq = conditional question which was only displayed/asked if applicable according to previous response patterns; of note, some conditional questions were classified as obligatory; t = total per section

### Participants and recruitments

In principle, the surveys were available for all hospital and community pharmacies in Germany, but they were invited in different ways.

All chief hospital pharmacists with membership within the ADKA (*n* = 345 from 358 German hospital pharmacies [27]) were invited by ADKA to participate via personal e-mail. For community pharmacies, no comprehensive list of e-mail addresses for all community pharmacies exist, and hence, a snowball approach was chosen for distribution of the survey. Thus, the Federal Union of German Associations of Pharmacists (ABDA) – as the national confederations of the 17 pharmacists’ associations and 17 German chambers of pharmacists – coordinated the invitation of distinct pharmacists via the 17 German federal chambers of pharmacists (aiming at all community pharmacies; however, all pharmacists working in Germany are compulsory members of one of the chamber of pharmacists (depending of where they live and work)). Depending on the respective chamber, different and often multiple invitation channels were used: seven chambers sent invitations to chief pharmacists via e-mail, three to every community pharmacist in their chamber, five displayed the link to the survey on their homepage, and eight included the link to the survey in web- or paper-based newsletters. At least two reminders were sent out via the same distribution channels. In addition, participation was encouraged via social media (via the authors´ private accounts, especially LinkedIn) and at the annual scientific congress of the ADKA which is also open for non-members.

To ensure that the surveys were still answered only once per pharmacy, this was pointed out in the introductory text of the survey and addressees were encouraged to pass on the access link to the employee who might be most suitable to answer the survey on the pharmacy`s behalf.

### Data collection

The surveys were open for 75 days, from April 17th to June 30th, 2023. The software LimeSurvey (Version 5.6.3, LimeSurvey GmbH, Hamburg, Germany) was used to present the self-administered online surveys and collect the respondents` answers. The respondents had access to the online survey in LimeSurvey via link or QR-Code sent as described above and entered their answers directly into it.

### Data analysis

The response patterns were extracted from LimeSurvey to Microsoft^®^ Excel^®^ 2019 (Redmond, USA), transferred to SPSS^®^ (IBM^®^ SPSS^®^ Statistics, Version: 28.0.0.0, Armonk, United States of America). Answer phrases were renamed in numbers for evaluation. All data were evaluated descriptively. Missing answers were not credited or excluded but reported. Entries in free text fields were not considered.

## Results

### Participants

Overall, 31.0% (111/358 [27]) of all German hospital pharmacies (Table 3) participated. Regarding the different chambers of pharmacists, the lowest response rates were observed in Bremen and Thuringia. Bremen was the single chamber of pharmacists of which no hospital pharmacy participated. The highest response rates were observed in Hamburg and Northrhine [27] (Additional file 2 – A1).

**Table 3:** Characteristics of participating hospital pharmacies

	Percentage (absolute numbers) of participating hospital pharmacies
Supplying other hospitals besides the main hospital with drugs	
yes	72.1% (80/111)
no	27.9% (31/111)
Number of beds in the main hospital	
less than 300 beds	5.4% (6/111)
300–600 beds	41.4% (46/111)
more than 600 beds	51.4% (57/111)
unable to assess	1.8% (2/111)
Location of the pharmacy	
urban	60.4% (67/111)
rural	39.6% (44/111)
Total number of pharmacists mainly and regularly working on wards (e.g. medical history-taking, supporting ward rounds, …)	2,6 *[mean]* (109/111)
no answer	1.8% (2/111)
More than half of the pharmacists have received certificates for successful participation in continuous education within the past three years	
yes	58.6% (65/111)
no	30.6% (34/111)
unable to assess	10.8% (12/111)
Employment of pharmacists in training	
yes – regularly (one pharmacist or more per year)	57.7% (64/111)
yes – irregularly (less than one pharmacist per year)	23.4% (26/111)
no	18.9% (21/111)
The person who answered the survey as a representative of the pharmacy was working in a hospital pharmacy before 2017	
yes	81.1% (90/111)
no	18.9% (21/111)

The presented socio-demographic data were surveyed as single-choice answers and/or free text input

4.5% (811/17,830 [28]) of all German community pharmacies (Table 4) participated. The lowest response rates were observed in Lower Saxony (0.4%) and Hamburg (1.6%), while the highest rates were reported for Mecklenburg Western Pomerania (8.6%) and Saxony-Anhalt (8.9%) [28] (Additional file 2 – A1).

In both cases, no clear trends were discernible depending on the region (e.g. North, West, East or South) or size of the chamber of pharmacists affiliated.

**Table 4:** Characteristics of participating community pharmacies

	Percentage (absolute numbers) of participating community pharmacies
The pharmacy is a member of a branch network	
yes – it is the main pharmacy	24.8% (201/811)
yes – it is the branch pharmacy	19.5% (158/811)
no	55.7% (452/811)
Total number of pharmacists currently working full-time	1.8 *[mean]* (714/811)
no answer	12.0% (97/811)
Total number of pharmacists currently working part-time (50% and more)	1.6 *[mean]* (563/811)
no answer	30.6% (248/811)
Total number of pharmacists currently working part-time (less than 50%)	1.2 *[mean]* (510/811)
no answer	37.1% (301/811)
More than half of the pharmacists have received certificates for successful participation in continuous education within the past three years	
yes	51.3% (416/811)
no	40.1% (325/811)
unable to assess	8.5% (69/811)
no answer	0.1% (1/811)
Location of the pharmacy	
urban	46.1% (374/811)
rural	53.9% (437/811)
Employment of pharmacists in training	
yes – regularly (one pharmacist or more per year)	8.9% (72/811)
yes – irregularly (less than one pharmacist per year)	54.5% (442/811)
no	36.0% (292/811)
no answer	0.6% (5/811)
Estimation of the ratio of regular customers to walk-in customers	
more regular customers	81.4% (660/811)
more walk-in customers	3.5% (28/811)
ratio is even	15.2% (123/811)
Number of patients per average day	
less than 70 patients	2.5% (20/811)
70–250 patients	76.7% (622/811)
more than 250 patients	20.8% (169/811)
Number of recently discharged patients (hospital discharge within the past week) per average month	
about one patient per month	5.1% (41/811)
1 – < 5 patient(s) per week	51.8% (420/811)
5–10 patients per week	30.7% (249/811)
> 10 patients per week	10.6% (86/811)
none	0.1% (1/811)
unable to assess	1.7% (14/811)
The person who answered the survey as a representative of the pharmacy was working in a community pharmacy before 2017	
yes	90.8% (736/811)
no	9.2% (75/811)

The presented socio-demographic data were surveyed as single-choice answers and/or free text input

### Impact of the new legal requirements on medication information transfer and medication supply

**Fig. 1:**
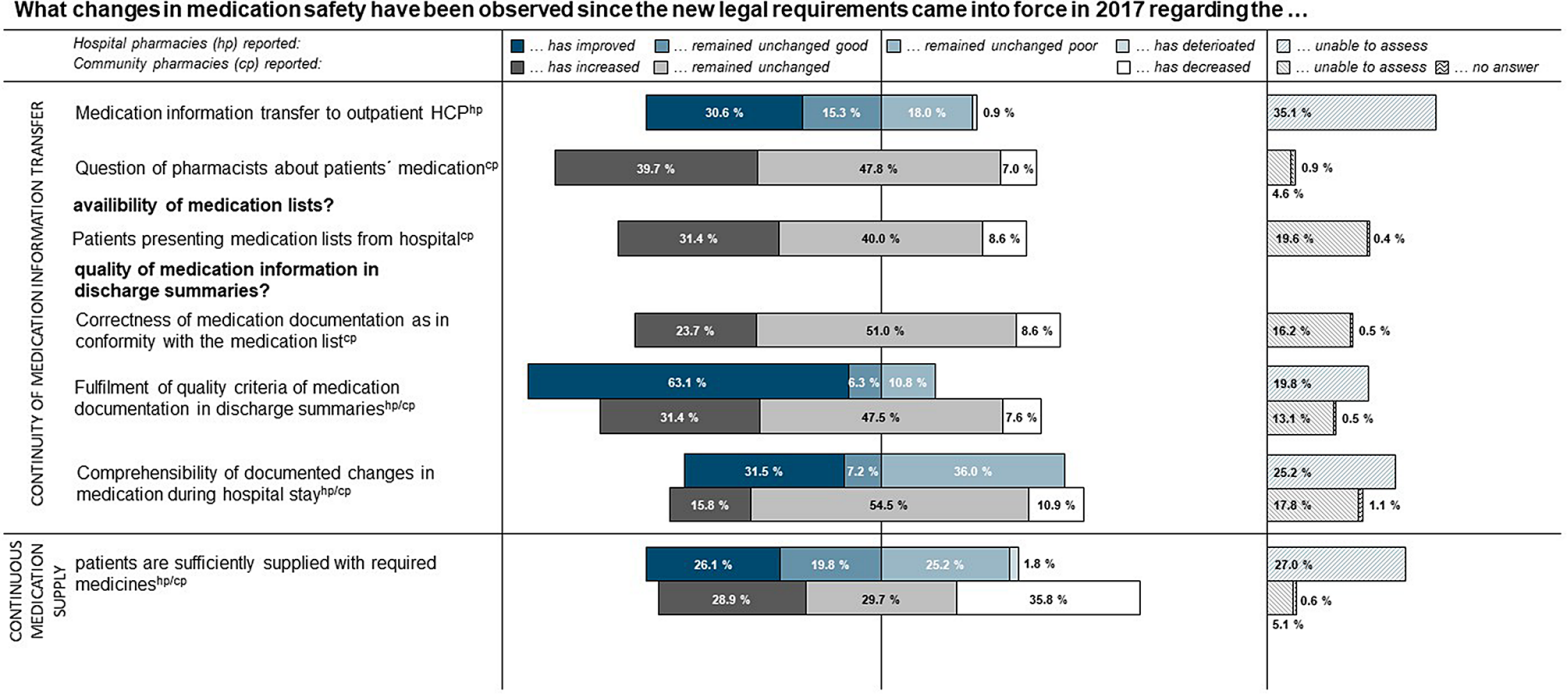
Content-orientated summary of responses on observed changes in medication safety since 2017. *This figure illustrates the content-orientated summary of the response patterns of the questions: (i) “What changes in medication safety and continuous* *medication supply* *have been observed over the past five years since the new legal requirements came into force? (single-choice answer (SC) per section; obligatory question for hospital pharmacies (hp) n = 111; community pharmacies (cp) n = 811) and (ii) “What changes in medication documentation in discharge summaries have been observed over the past five years since the new legal requirements came into force?” (SC per section; community pharmacy n = 659). For better comparability and comprehensibility, the original wordings are partly paraphrased, the responses of the community pharmacies to “Patients who lack the required medication” are presented in reverse order. Only selected items are presented*

About one-third (31.4%; 207/659) of the community pharmacies, that regularly saw discharge summaries, reported improvements in the quality of medication documentation since the introduction of the new legal requirements. Nearly half of them indicated that they have perceived no changes (47.5%; 313/659) or even deteriorations (7.6%; 50/659). In comparison, 63.1% (70/111) of the hospital pharmacies perceived the quality as improved while 17.1% (19/111) described it as unchanged or deteriorated. In terms of the comprehensibility of the documentation of inpatient changes in medication, half of the community pharmacies (54,5%; 359/659) perceived no changes and 15.8% an improvement (104/659). In comparison, 31.5% (35/111) of the hospital pharmacies observed an improvement and 36.0% (40/111) rated it as unchanged poor (Fig. 1). Regarding continuous medication supply, 35.8% (290/811) of the community pharmacies observed an increasing number of patients lacking the required medication during hospital-to-home transitions, while 29.7% (241/811) reported no changes. In comparison, 1.8% (2/111) of the hospital pharmacies rated the numbers as increased, while 45.0% (50/111) had not noticed any changes (Fig. 1).

### Continuity of medication information transfer

#### Medication lists

**Fig. 2:**
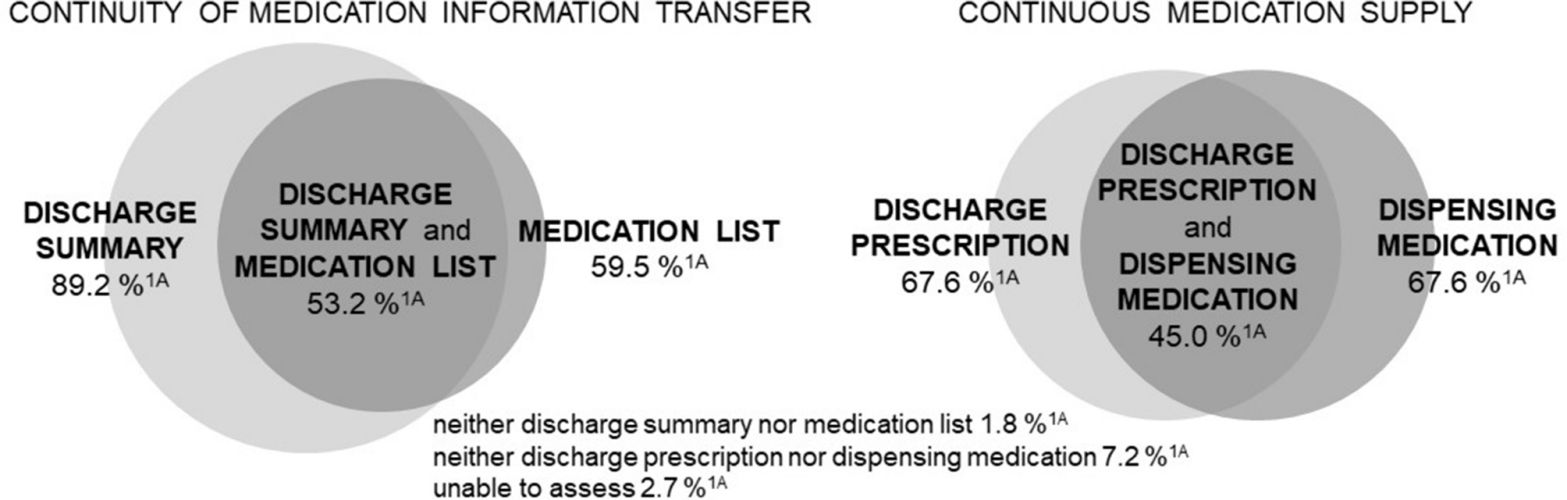
Hospital pharmacies´ perceptions of the implementation of discharge summaries, medication lists, discharge prescriptions and dispensing medication in routine care. ^1A ^Proportion of hospitals typically handing over the respective documents/medications stated by participating hospital pharmacies (percentage of n = 111). This figure illustrates the response pattern of the question: “What is typically handed over to patients at hospital discharge?” (multiple-choice answer (MC); n = 111)

Almost two in three hospital pharmacies (59.5%; 66/111) indicated that patients typically received medication lists from hospital at discharge (Fig. 2). Conversely, nearly half of the community pharmacies (49.0%; 390/796) reported that about or less than half of recently discharged patients presented medication lists from hospitals upon request (Additional file 2 – A2).

About half of the participating community pharmacies observed insufficient comprehensibility of medication lists for patients (54.7%; 352/643) and/or discrepancies between medication lists and further medication documentation such as discharge summaries (49.8%; 320/643). Furthermore, most community pharmacies (73.4%; 472/643) indicated that inpatient healthcare professionals could not be reached in time in case of queries (Additional file 2 – A3).

From an inpatient perspective, shortages in time/personal resources (77.8%; 14/18) and/or technical issues (50.0%; 9/18) were named most frequently as difficulties by hospital pharmacies, who stated to be actively involved in preparing and/or issuing medication lists as part of discharge processes (Additional file 2 – A3).

#### Discharge summaries

About nine in ten hospital pharmacies indicated that patients were typically provided with discharge summaries when leaving the hospital (89.2%; 99/111) (Fig. 2). Nearly every second of the community pharmacies (48.7%; 388/796) estimated that they received a discharge summary less than once per week (Additional file 2– A2).

Besides, the majority of hospital pharmacies that actively participated in composing discharge summaries described most frequently limited time and personnel resources as barriers (63.6%; 7/11) (Additional file 2 – A3).

### Continuous medication supply

One-third of the community pharmacies (31.0%; 247/796) indicated that about half or more recently discharged patients lacked any required medication during hospital-to-home transitions (Additional file 2 – A2).

#### Discharge prescriptions

Issuing discharge prescriptions was typically used according to 67.6% (75/111) of hospital pharmacies (Fig. 2). Nearly one-third of the community pharmacies (27.6%; 220/796) experienced that more than half of recently discharged patients presented discharge prescriptions. The majority of those community pharmacies estimated that more than half of these discharge prescriptions could be filled in and medication dispensed in time of the next administration (73.3%; 448/611) (Additional file 2– A2). However, half of the community pharmacies (50.1%; 306/611) reported that medication documentation of discharge prescriptions was incomplete or ambiguous. Furthermore, they indicated poor compliance with formal requirements – such as prescribing the smallest package size (62.5%; 382/611), using institutional identification (48.8%; 298/611), the obligation to be issued by specialists (31.8%; 194/611), using the correct template (24.5%; 150/611) and others (e.g. missing dosage) (75.1%; 459/611). They reported difficulties in accessing inpatient healthcare professionals in a timely manner to clarify queries (79.2%; 484/611) and in the availability of prescribed active substances (65.1%; 398/611) and package sizes (60.7%; 371/611) in the German market. Besides that, 68.7% (420/611) of community pharmacies indicated that patients presented expired discharge prescriptions.

From an inpatient perspective, over half of the hospital pharmacies that prepared and/or supported issuing discharge prescriptions reported technical issues (55.0%; 11/20) and/or lack of time/personnel resources (60.0%; 12/20) (Additional file 2 – A3).

#### Dispensing required medication at patients´ discharge

According to 67.6% (75/111) of the hospital pharmacies, patients were typically provided with the required medication by dispensing selected medication at hospital discharge, if needed (Fig. 2). Half of the hospital pharmacies that stated they were actively involved indicated that it was difficult to adequately inform patients about received medications (50.0%; 11/22) (Additional file 2 – A3).

### Facilitators to implement the new legal requirements in inpatient routine care

The majority of the hospital pharmacies indicated that the implementation of the new legal requirements was beneficially supported by software-based medication documentation (62.2%; 69/111). Over one-third named successful interprofessional communication as a facilitator (36.0%; 40/111). Further facilitators were reported less frequently – such as increasing the number of pharmaceutical staff (16.2%; 18/111), support of the hospital´s board of directors (15.3%; 17/111), and increasing the number of further staff (9.9%; 11/111). About one in seven (14,4%; 16/111) hospital pharmacies indicated that there were no facilitators (Additional file 2 – A4).

## Discussion

The two surveys provide initial insights into how hospital and community pharmacists perceive medication information transfer and medication supply during hospital-to-home transitions. According to their assessment, recently discharged patients were still lacking medication lists and/or discharge summaries. Moreover, even if these documents were handed over, the quality in terms of completeness, correctness and understandability was not guaranteed. Furthermore, pharmacists indicated that some patients were still insufficiently supplied with medication immediately after discharge. As these observations were made even five years after legal requirements had been set to address these issues, it can be assumed that these might not be fully and sustainably implemented as intended in daily practice of all hospitals. Moreover, it is also worth discussing whether the requirements as such are suitable for meeting the intended purposes.

### Continuity of medication information transfer

Medication lists are crucial for communicating medication information to patients, while discharge summaries are still the primary mean of communication at discharge. Both must be handed over to patients at discharge [13]. Though, four in ten hospital pharmacies indicated that patients were not typically provided with medication lists, only 1.8% of the hospital pharmacies stated that patients typically received neither medication lists nor discharge summaries. As the latter are originally addressed to health care professionals, comprehensibility for patients is not guaranteed. Hence, it has to be assumed that there were still patients lacking understandable written medication information. While the discharge summaries originally address primary care physicians, also nearly half of the community pharmacies reported to receive discharge summaries less than once a week, while nearly all (94.8%) reported to see at least one recently discharged patient per week. For future studies it would be interesting to complement these results by assessing the process of issuing and receiving discharge summaries also with hospital and primary care physicians.

About every second community pharmacy indicated that medication lists from hospitals were presented upon request by only half or fewer of the recently discharged patients. This was roughly consistent with previous evaluations in Germany, which found that 72% of patients who take medication have medication lists, and 57% present these at hospital admissions [29].

In addition to the mere availability of these documents, their completeness, correctness and comprehensibility are crucial. This is particularly important as nearly three-quarters of the community pharmacies (73.4%) described poor accessibility of inpatient healthcare professionals in case of queries. Additionally, medications often change during hospitalisation as medications are discontinued or newly prescribed [4]. Indeed, one in two community pharmacies reported medication discrepancies between medication lists and other documents (e.g. discharge summary) (49.8%) and poor patients´ comprehensibility of medication lists (54.7%). This also makes it more difficult for patients to self-manage their medication when discharged from hospital.

Previous quality assessments of medication lists have also shown that none of the documents analysed were complete and 79% did not comply with the essential criteria of medication documentation [10, 30]. Even more, medication discrepancies seem to be common problems after hospital discharge [30–33]. Even if explanation of changed medications are required to be documented in discharge summaries by the new legislation and primary care physicians as recipients explicitly request those [34, 35], only 15.8% of the community pharmacies and 31.5% of the hospital pharmacies perceived an improvement and another aspect that might be particularly challenging for patients, e.g. the switch between different brand names triggered by the hospital formulary, is not even considered in this regard. However, it is suggested to document medication in one cross-sectional system that is curated by (or at least visible to) both in- and outpatient health care professionals. Thus, information flow might improve and information on medication (changes) might become easier available [36–38]. Using such overarching systems would also facilitate the implementation of new standards in documentation of medicinal products such as ISO Identification of Medicinal Products [39].

### Continuous medication supplies

According to a third of the community pharmacies (31.0%), about half or more patients still lacked required medication during hospital-to-home transitions. Unexpectedly, 35.8% community pharmacies stated that this number has increased since the introduction of the new legal requirements. In contrast, 67.6% of the hospital pharmacies indicated that patients received discharge prescriptions if needed. If these were received by community pharmacies in time, about 73.3% indicated that more than half could be dispensed. Nonetheless, community pharmacies often reported poor compliance with formal requirements or ambiguous medication documentation of discharge prescriptions. This can cause delays in dispensing or prevent a prescription from being filled. This might also happen due to poor availability of prescribed active substances or package sizes in the German market as these were reported by two thirds of the community pharmacies. Besides that, the majority of community pharmacies experienced that patients presented expired discharge prescriptions (primary non-compliance). This is a common issue and has been shown to cause adverse outcomes [40–42].

### Perspectives

The hospital pharmacies identified structured medication documentation via software and interprofessional communication as facilitators for the implementation of the new legal requirements. New technical developments addressing these requests are about to be launched in Germany within the next years. They will enable healthcare professionals to electronically exchange (bidirectional) medication information in a structured format – such as medication lists, discharge summaries, queries, or even text messages for more urgent matters [43, 44]. However, the electronic exchange of medication information by itself might not guarantee to sustainably close communication gaps as indicated by a Swedish observational study [45]. Thus, even with those soon-to-be new options in Germany, it is important for healthcare professionals to use them in compliance with the purpose of an effective discharge management and to further assess their impacts on medication safety before adding further supportive interventions. Those could be structured pharmacist-led medication reconciliations during hospital-to-home transitions. There is evidence that these may positively impact medication-related readmissions [46, 47]. Pharmacist-led medication reconciliations may either be conducted pre-discharge as pharmacists review medication lists in discharge summaries and the communication of changes in medication [48] or post-discharge as community pharmacies directly receive discharge medication information from hospital [49, 50].

### Strengths and limitations

To the best of our knowledge, these are the first surveys asking all hospital and community pharmacies in Germany about their perceptions of hospital-to-home transitions with regard of continuity of medication information transfer and continuous medication supply. The response rate of 31.0% (111/358) of all German hospital pharmacies seemed to be reasonably sufficient and rather common in comparison to experiences from e.g. the United States [51, 52]. In comparison, the proportion of participating community pharmacies (4.5%) seemed to be relatively small. However, for example, a Swedish online survey (of pharmacies´ employees; invitations distributed via e-mail by the main owning companies) reached a response rate of 5% (228/4900) [53]. In relation to this experience, our response rate of 4.5% (811/17,830) seemed to be comparable and even higher compared to a German survey study. They invited community pharmacists of five chamber of pharmacists via e-mail and received at least partly responses of 141 community pharmacists [54] of roughly 8100 community pharmacies [28]. Nevertheless, bias due to the rather small sample size cannot be ruled out. Furthermore, the response rates differed between the different chamber of pharmacists that might also limit the generalisability. Though, we have not observed particular tendencies between the response rates depending on the region (e.g. North, West, East or South) nor the size of the chamber of pharmacists. The differences in response rates between hospital and community pharmacies might have been also influenced by the different invitation channels used. While nearly all chief hospital pharmacists were invited to participate via personal e-mail, the community pharmacies were invited on different and often multiple ways (e.g. e-mail to chief pharmacists and/or every community pharmacist, displayed access data on their homepage, web- or paper-based newsletters) depending on the respective chamber of pharmacists.

Moreover, hospital and community pharmacies were not technically linked or matched and might have varying views on a specific care process. In favour of anonymity, it could not be technically prevented from answering the surveys twice by the same person or different employees of one pharmacy. However, the chance was rather unlikely as we emphasised in the surveys´ invitations and introductions that respondents should only answer once per pharmacy, the surveys as such caused a rather high expenditure of time and only deliberately submitted responses were included in the evaluation.

In addition, we asked for rather general assessments and estimations of current routine care as closed-ended questions than precise numbers (which are indeed difficult to set into a context). Thus, the results are an approach to actual routine care. Conditional questions ensured that only applicable questions were displayed to specific groups of respondents – resulting in fewer evaluable responses for most of the questions of hospital pharmacies. Furthermore, the study did not differentiate in the specialities of the respective hospitals or similar.

As the intention of this survey study was to provide first insights into medication safety during hospital-to-home transitions from the pharmacists’ perspective, further studies are needed to include perspectives from other in- and outpatient healthcare professionals like general practitioners as well as patients to provide a more comprehensive understanding. In addition, as it has to be assumed that there might be still insufficient medication information transfers and lacks in medication supply in today´s routine care, the extent of these risks and their actual impact on medication safety should be further investigated.

## Conclusion

The new legal requirements were originally intended to improve the continuity of medication information transfer and continuous medication supply during hospital-to-home transitions. Five years after introducing our survey study suggests that implementation of these requirements into daily practice might still be deficient and risks of medication-related harm still remain. Potentially, three implementation gaps could be distinguished – firstly, adoption rate of the new requirements seemed below 100%, secondly, quality of the implementation might not always be sufficient and third, even if implemented, the requirements might not be sufficient to close all gaps in information transfer and medication supply. Given Germany’s currently changing digital infrastructure, a close look is recommended to assess how future digital care processes will facilitate discharge management and support medication safety.

### Electronic supplementary material

Below is the link to the electronic supplementary material.

Supplementary Material 1: Additional File 1 presents the non-validated translations of the applied surveys.

Supplementary Material 2: Additional File 2 presents the full response patterns to the questions.

## Data Availability

Both surveys were developed for the presented study and conducted in German language. Non-validated translations of these surveys are provided in the Additional file 1. To use these surveys or parts of them, please contact klinische.pharmakologie@med.uni-heidelberg.de for permission. The originally used survey templates are available upon resonable request. All data analysed in this paper are presented within the paper, its figures, tables and supplementary information files.
