# Supplementary material for: Continuity of medication information transfer and continuous medication supply during hospital-to-home transitions - nationwide surveys in hospital and community pharmacies after implementing new legal requirements in Germany
Source: BMC Health Serv Res. 2024 Aug 27;24:993. doi: 10.1186/s12913-024-11208-4 (PMC11348592; doi:10.1186/s12913-024-11208-4)
Supplement: Supplementary file 1 — Supplementary Material 1: Additional File 1 presents the non-validated translations of the applied surveys. [file 12913_2024_11208_MOESM1_ESM.pdf]

Additional file 1 – non-validated translations of the applied surveys

Continuity of medication information transfer and continuous medication supply at hospital-to-home-transitions - nationwide surveys in hospital and community pharmacies after implementing new legal requirements in Germany

Sophia Klasing [1,2], Frank Dörje [2,3], Heike Hilgarth [2,4], Nadine Metzger [2,5], Ina Richling [2,6,7], Hanna M. Seidling [1,2] *

* Correspondence

Hanna M. Seidling

Hanna.seidling@med.uni-heidelberg.de

The surveys were developed for the presented study and conducted in German language. This document presents a non-validated translation into English that was produced as part of the submission of the manuscript. During the study, the survey was administered as dynamic online survey while this document only lists the questions and answer-schemes statically and in a non-formatted way.

To use these surveys or parts of them, please contact klinische.pharmakologie@med.uni-heidelberg.de for permission. The originally used survey templates in German are available upon reasonable request.

Implementation of and experiences with the discharge management from hospital pharmacies´ perspective

1. General and sociodemographic questions

1.1. Does your pharmacy supply other hospitals besides the main hospital with drugs? (Single-choice question)

○ Yes

○ No

1.2. How many beds does your pharmacy supply in your hospital? (Single-choice question)

○ Less than 300 beds

○ 300 – 600 beds

○ More than 600 beds

○ Unable to assess

1.3. In which chamber-of-pharmacist district is your pharmacy located? (Single-choice question)

○ Bremen

○ Hamburg

○ Berlin

○ Saarland

○ Schleswig Holstein

○ Thuringia

○ Saxony

○ Rhineland Palatinate

○ Saxony-Anhalt

○ Hesse

○ Mecklenburg Western Pomerania

○ Brandenburg

○ Northrhine

○ Westphalia-Lippe

○ Baden-Wuerttemberg

○ Lower Saxony

○ Bavaria

1.4. Is the location of your pharmacy … ? (Single-choice question)

○ Urban

○ Rural

1.5. How many of the pharmacists of your pharmacy mainly and regularly work on wards (e.g. medical history-taking, supporting ward rounds, …)? (Freetext input)

1.6. Have more than half of the pharmacists of your pharmacy received certificates for successful participation in continuous education within the past three years? (Single-choice question)

○ Yes

○ No

○ Unable to assess

1.7. Does your pharmacy employ and educate pharmacists in training? (Single-choice question)

○ Yes – regularly (one pharmacist or more per year)

○ Yes – irregularly (less than one pharmacist per year)

○ No

1.8. Have you personally worked in a hospital pharmacy before 2017? (Single-choice question)

○ Yes

○ No

2. General questions about the new legal requirements (e.g. current barriers to implementation)

2.1. Who was involved in conceptualising the implementation of the new requirements of the discharge management in your hospital (e.g. development and implementing local standard operating procedures)? (Multiple-choice question)

- O Pharmaceutical staff of the hospital pharmacy
- O Physicians of certain specialities of the hospital
- O Physicians of all specialities of the hospital
- O Nurses
- O Staff of the patient management
- O Social workers
- O Staff of the quality/risk management
- O Computer scientists
- O Unable to assess
- O Others: (Text input option)

In the following part of the survey, the wording *"the new legal requirements"* refers to [1]:

- Handing out discharge summaries (at least as a preliminary version) at patients´ discharge from hospital
- Handing out medication lists (e.g. federal standard medication plan) for patients at their discharge
- After assessing the patients´ supply with required provision, if needed, discharge prescriptions are issued or medication dispensed

2.2. How are the new legal requirements implemented in the hospital? (Single-choice question)

- O There are guidelines which apply to the entire hospital. (e.g. uniform standard operating procedures for the entire hospital)
- O There are guidelines which apply to certain divisions of the hospital. (e.g. standard operating procedures for internal medicine or surgery)
- O There are no internal guidelines
- O Unable to assess

- Others: (Text input option)

2.3. What changes in medication safety and continuous medication supply have been observed over the past five years since the new legal requirements came into force? (Single-choice question)

For each response item (2.3.a – 2.3.f) it should be chosen between: (i) has improved (ii) remained unchanged good (iii) remained unchanged poor (iv) has deteriorated (v) unable to assess.

- Fulfilment of quality criteria of medication documentation in discharge summaries (e.g. drug name, strength, dosage regimes, …)
- Comprehensibility of documented changes in medication during hospital stay
- Availability of medication information for all involved health care professionals (e.g. pharmacists) during hospital stay
- Medication information transfer to outpatient health care professionals
- Patients´ knowledge about their current medication
- Number of patients who are sufficiently supplied with required medicines

2.4. Are internal process measures implemented at the hospital which assess whether the new legal requirements are implemented correctly?

- Yes – there are regular assessments
- Yes – there are irregular assessments
- No – there are no internal process assessments
- Unable to assess
- Others: (Text input option)

2.5. Are you using software for structured medication documentation in scope of discharge management? (Multiple-choice question)

- Yes, for medication lists
- Yes, for medication documentation in discharge summaries
- Yes, for discharge prescriptions
- No

○ Unable to assess

○ Others: (Text input option)

○ If yes, which Software are you using (Text input option)

2.6. What is typically handed over to patients at hospital discharge? (Multiple-choice question)

○ Medication list

○ (preliminary) discharge summary

○ Discharge prescription if needed

○ Patients are not provided with any written medication-related documents

○ Selected medication if needed

○ Unable to assess

○ Others: (Text input option)

2.7. What are currently facilitators to implement the new legal requirements in inpatient routine care? (Multiple-choice question)

○ Software-based medication documentation processes

○ Successful interprofessional communication

○ Increased numbers of pharmaceutical staff

○ Increased number of further staff

○ Support of the hospital´s board of directors

○ There are no facilitators

○ Unable to assess

○ Others: (Text input option)

2.8. What are currently barriers to implement the new legal requirements in inpatient routine care?

For each response item (2.8.a – 2.8.f) there were multiple-choice options: (i) no difficulties (ii) difficulties in organising processes (iii) technical issues (iv) shortage in staff/ timely resources (v) unable to assess has improved.

○ Providing patients with up-to-date medication lists at discharge

- Providing patients with (at least preliminary) discharge summaries at discharge
- Fulfilment of quality criteria of medication documentation in discharge summaries
- Comprehensibility of documented changes in medication during hospital stay
- Issuing discharge prescriptions
- Dispensing required medicines at discharge

3. Regular involvement of pharmaceutical staff in clinical processes, including discharge management

3.1. What pharmaceutical activities are currently regularly performed on wards by your pharmacy?

For each response item (3.1.a – 3.1.i) it should be chosen between: (i) regularly for the majority of wards (ii) regularly for the minority of wards (iii) irregularly on demand (iv) not involved but would like to implement it (v) not involved and would not like to implement it.

- Involvement of pharmaceutical staff in hospital admissions (e.g. medical history-taking, adapting pre-admission medication to typical inpatient medicines provision, …)
- Involvement of pharmaceutical staff in ward rounds (e.g. in person or only patients´ medical-record screening)
- Medication reviews in in the context of the discharge planning
- Preparation and/or compilation of medication lists to be handed out to patients at discharge
- Pharmaceutical patient consultation in the context of discharge (e.g. including trainings)
- (Supporting) assessment of actual patients´ need for medication supply
- Issuing discharge prescriptions and/or supporting it
- Preparation of dispensing drugs upon discharge and/or supporting it
- Supporting issuing discharge summaries (e.g. preparing medication documentation; excluding only transferring pharmaceutical advices made during ward rounds)

4. Medication information transfer – medication list

4.1. How many pharmacists are involved in compiling medication lists on average per workday? (Text input option as number of pharmacists per workday)

4.2. For how many wards are the pharmacists performing that? (Text input option as number of wards per week)

4.3. How many minutes are required on average per patient (including preparation, follow-up and documentation)? (Text input option as minutes per patient)

4.4. How is the medication documented on the wards? (Single-Choice question)

- ○ Digital documentation in the entire hospital
- ○ Digital documentation on the majority of wards – paper-based documentation on the minority of wards
- ○ Digital documentation on the minority of wards – paper-based documentation on the majority of wards
- ○ Paper-based documentation in the entire hospital
- ○ Unable to assess
- ○ Others: (Text input option)

4.5. How are the medication information transferred from the medication documentation on the wards to the medication lists? (Single-Choice question)

- ○ Mainly via unstructured text input into the software which documents medication lists
- ○ Mainly via structured entry fields into the software which documents medication lists
- ○ Mainly (partly) automatically via transfer function of electronic prescription software
- ○ Unable to assess
- ○ Others: (Text input option)

4.6. What format is used for medication lists to be handed over to patients? (Single-Choice question)

- ○ Mainly paper-based federal standard medication plan
- ○ Mainly paper-based medication list in an individual format
- ○ Mainly electronic federal standard medication plan (e.g. to be transmitted via app)

- ○ Mainly electronic medication list in individual format (e.g. to be transmitted via app)
- ○ Mainly no medication lists are provided
- ○ Unable to assess
- ○ Others: (Text input option)

4.7. What difficulties in compiling medication lists are currently observed in routine care? (Multiple-Choice question)

- ○ Difficulties in timely availability of medication information
- ○ Missing or incomplete comparison with pre-admission medication and adaptions to the medication lists if needed
- ○ Technical issues (e.g. transferring medication information from medication documentation on wards)
- ○ Difficulties in inpatient interprofessional and/or interdisciplinary communication (e.g. in case of queries)
- ○ Shortage in time and staff resources
- ○ No difficulties
- ○ Unable to assess
- ○ Others: (Text input option)

5. Medication information transfer – patient consultation

5.1. How many pharmacists are involved in patient consultation in scope of discharge management on average per workday? (Text input option as number of pharmacists per workday)

5.2. For how many wards are the pharmacists performing that? (Text input option as number of wards per week)

5.3. How many minutes are required on average per patient (including preparation, follow-up and documentation)? (Text input option as minutes per patient)

5.4. About what type of medication do you talk about typically in those “patient consultations” with patients and/or their relatives or informal caregivers? (Multiple-Choice question)

- ○ Newly prescribed medication

- Medication that was changed or ceased during hospital stay
- Drugs of certain therapeutic groups (e.g. anticoagulants, antibiotics, anticancer drugs)
- Drugs with special application forms (e.g. inhaler, pens, syringes)
- Medication that is handed over to patients at discharge
- No medication in particular or rather always different medication
- Unable to assess
- Others: (Text input option)

5.5. About what drug-related issues do you talk about typically in those “patient consultations” with patients and/or their relatives or informal caregivers? (Multiple-Choice question)

- Dosage regimes
- Preparation of medication to be administered (e.g. dividing tablets)
- Instructions on how to administer special application forms (e.g. inhaler)
- Advices for administration (e.g. do not administer L-Thyroxin together with Calcium)
- Treatment duration
- Indication and potential benefits of the treatment
- Potential drug-drug interactions with further medication
- Potential adverse drug events
- Changes in medication which were made during hospital stay
- Storage of drugs at home
- Advices for drug monitoring (e.g. blood pressure)
- Unable to assess
- Others: (Text input option)

5.6. What drug-related problems do you currently observe in patients whose discharge is prepared? (Selection and wording of drug-related problems based on [2]) (Multiple-choice question)

- Drug-drug interactions
- Adverse drug events

- Unnecessary duplications of medication (e.g. drugs with the same active ingredients and/or of the same therapeutic group)
- Inappropriate dosing intervals (e.g. Administration of an antibiotic at 8am, 12am and 6pm instead of every 8 hours)
- Inappropriate dosage regimes (e.g. in case of renal impairment)
- Inappropriate treatment duration (e.g. short-term medication is prescribed as long-term treatment)
- Inappropriate dose timing (e.g. taking diuretics in the evening)
- Inappropriate drug selection (e.g. medication in discordance with guidelines)
- Inappropriate application form (e.g. powder inhaler for patients with weakened breathing)
- Difficulties in administering the medication (e.g. applicating inhaler or dividing tablets)
- Contraindications (e.g. due to comorbidities, allergies or age)
- Drug-food interactions (e.g. grape fruit)
- Overuse (e.g. medication without indication)
- Underuse (e.g. indication without medication)
- Patients´ lack of knowledge about new or changed medication
- Non-adherence
- We do not observe drug-related problems
- Unable to assess

5.7. What difficulties are currently observed in patient consultation in routine care? (Multiple-choice question)

- Difficulties in communication with patients (e.g. comprehension difficulties and failing linguistic ability)
- Limited patients´ capacity to comprehend medication information at discharge
- Difficulties in timely availability of medication information
- Missing communication on who is going to be discharged and when
- Difficulties in inpatient interprofessional and/or interdisciplinary communication

- ○ Shortage in time and staff resources
- ○ No difficulties
- ○ Unable to assess
- ○ Others: (Text input option)

6. Continuous medication supply – discharge prescriptions

6.1. How many pharmacists are involved in issuing discharge prescriptions and/or supporting it on average per workday? (Text input option as number of pharmacists per workday)

6.2. For how many wards are the pharmacists performing that? (Text input option as number of wards per week)

6.3. How many minutes are required on average per patient (including preparation, follow-up and documentation)? (Text input option as minutes per patient)

6.4. Are you able to approximately estimate how many patients are discharged with discharge prescriptions? (Single-choice question)

- ○ Yes
- ○ No

6.5. To what proportion of discharged patients are discharge prescriptions handed out? (Text input option)

6.6. What difficulties are currently observed in issuing discharge prescriptions in routine care? (Multiple-choice question)

- ○ Technical issues in compiling the discharge prescriptions (e.g. software, printing)
- ○ Missing or inappropriate compliance to the obligation to be issued by senior physician
- ○ Missing or inappropriate compliance to the smallest packages
- ○ Missing or inappropriate compliance to further requirements (e.g. documenting dosage regimes, signatures, …)
- ○ Difficulties in timely availability of medication information
- ○ Difficulties in assessing the actual patients´ need for medication supply
- ○ Difficulties in inpatient interprofessional and/or interdisciplinary communication
- ○ Shortage in time and staff resources

- ○ No difficulties
- ○ Unable to assess
- ○ Others: (Text input option)

6.7. How is the supply with narcotics organized? (Single-choice question)

- ○ Mainly the hospital physicians have discharge prescription templates for narcotics and after issuing those, the narcotics are dispensed by the hospital pharmacy
- ○ Mainly the hospital physicians have discharge prescription templates for narcotics and after issuing those, the narcotics are not dispensed by hospital pharmacy
- ○ Mainly a consultation service (e.g. pain outpatient clinic) issues discharge prescription of narcotics
- ○ No discharge prescriptions of narcotics are issued, instead due to timely communication, patients are supplied by general practitioners or emergency services
- ○ There is no uniform process structure
- ○ Unable to assess
- ○ Others: (Text input option)

7. Continuous medication supply – dispensing drugs upon discharge

7.1. How many pharmacists are involved in dispensing drugs upon discharge on average per workday? (Text input option as number of pharmacists per workday)

7.2. For how many wards are the pharmacists performing that? (Text input option as number of wards per week)

7.3. How many minutes are required on average per patient (including preparation, follow-up and documentation)? (Text input option as minutes per patient)

7.4. In what occasions is dispensing drugs upon discharge typically preferred? (Multiple-choice question)

- ○ To end drug treatments which have been started during hospital stay
- ○ Medications which are newly prescribed during hospital stay
- ○ Medications which dosage regimes have been changed during hospital stay

- Medications of certain therapeutic groups (e.g. anticoagulants, antibiotics, anticancer drugs)
- Mainly all medications
- Patients with limited mobility
- Patients with cognitive impairment
- Patients who are not able to visit outpatient physicians in time for further prescriptions
- Patients who have not a family practitioner
- Before weekends and public holidays
- There is no special preference of special occasions or rather always different
- Unable to assess
- Others: (Text input option)

7.5. To what extant are drugs typically dispensed upon discharge? (Multiple-choice question)

- Needed supply for one day
- Needed supply for following weekends and/or public holiday
- Only the needed supply of selected medications
- Unable to assess
- Others: (Text input option)

7.6. How are drugs typically dispensed upon discharge? (Multiple-choice question)

- The personalised drug day-dispenser used on wards is handed out (e.g. including single tablets) – without additional labelling of the medication by hospital staff
- Drugs in the primary package (e.g. tablets in blisters without packages)
- With additional (individual) labelling of the medication by hospital staff
- Within a personalised blister as part of unit-dose-packages provided by the hospital pharmacy
- With package leaflet
- Unable to assess
- Others: (Text input option)

7.7. How are patients typically informed about dispensed drugs upon discharge (e.g. about dosage regimes, advices for administration, indication, …)? (Multiple-choice question)

- ○ Orally e.g. as part of patients' consultation with hospital pharmacists
- ○ Written via handing out package leaflets or respective QR-codes
- ○ Written via medication lists
- ○ Written via documentation on packages
- ○ Mainly e.g. hospital physicians or nurses inform patients orally
- ○ There is no uniform process structure
- ○ Unable to assess
- ○ Others: (Text input option)

7.8. What difficulties are currently observed in dispensing drugs upon discharge in routine care? (Multiple choice question)

- ○ Insufficient information of patients about dispensed drugs
- ○ Timely availability of medication information
- ○ Difficulties in inpatient interprofessional and/or interdisciplinary communication
- ○ Patients are not able to recognize dispensed drugs in their current medication
- ○ Difficulties in assessing the actual patients´ need for medication supply
- ○ Shortage in time and staff resources
- ○ No difficulties
- ○ Unable to assess
- ○ Others: (Text input option)

8. Medication information transfer – supporting issuing discharge summaries

8.1. How many pharmacists are involved in supporting issuing discharge summaries on average per workday? (Text input option as number of pharmacists per workday)

8.2. For how many wards are the pharmacists performing that task? (Text input option as number of wards per week)

8.3. How many minutes are required on average per patient (including preparation, follow-up and documentation)? (Text input option as minutes per patient)

8.4. How are the medication information transferred from the medication documentation on the wards to the medication documentation in discharge summaries? (Single-Choice question)

- ○ Mainly via unstructured text input into the software which documents discharge summaries
- ○ Mainly via structured entry fields into the software which documents discharge summaries
- ○ Mainly (partly) automatically via transfer function of electronic prescription software
- ○ Unable to assess
- ○ Others: (Text input option)

8.5. Are pharmaceutical advices which were made during ward rounds documented in discharge summaries? (Single-choice question)

- ○ Yes
- ○ No
- ○ Unable to assess

8.6. What difficulties in supporting issuing discharge summaries are currently observed in routine care? (Multiple-Choice question)

- ○ Technical issues (e.g. software, printing)
- ○ Difficulties in timely availability of medication information
- ○ Difficulties in inpatient interprofessional and/or interdisciplinary communication (e.g. in case of queries)
- ○ Missing or incomplete comparison with pre-admission medication and adaptions to the medication lists if needed
- ○ Shortage in time and staff resources
- ○ No difficulties
- ○ Unable to assess
- ○ Others: (Text input option)

9. Potential for improvement of discharge processes

9.1. What further interventions to improve medication safety are you performing routinely or in scope of projects? (Text input option)

9.2. How could you as hospital pharmacy better support patient after hospital discharge (Text input option)

9.3. What would you need to do so? (Text input option)

Implementation of and experiences with the discharge management from community pharmacies´ perspective

1. General and sociodemographic questions

1.1. Is your pharmacy member of a branch network? (Single-choice question)

○ Yes – it is the main pharmacy

○ Yes – it is the branch pharmacy

○ No

1.2. How many pharmacists are currently working in your pharmacy?

○ Working full-time (Number as text input option)

○ Working part-time (50 % and more) (Number as text input option)

○ Working part-time (less than 50 %) (Number as text input option)

1.3. Have more than half of the pharmacists received certificates regarding successful participation in continuous education within the past three years? (Single-choice question)

○ Yes

○ No

○ Unable to assess

1.4. In which chamber-of-pharmacist district is your pharmacy located? (Single-choice question)

○ Bremen

○ Hamburg

○ Berlin

○ Saarland

○ Schleswig Holstein

○ Thuringia

○ Saxony

○ Rhineland Palatinate

○ Saxony-Anhalt

○ Hesse

○ Mecklenburg Western Pomerania

○ Brandenburg

○ Northrhine

○ Westphalia-Lippe

○ Baden-Wuerttemberg

○ Lower Saxony

○ Bavaria

1.5. Is the location of your pharmacy … ? (Single-choice question)

○ Urban

○ Rural

1.6. Does your pharmacy employ and educate pharmacists in training? (Single-choice question)

○ Yes – regularly (one pharmacist or more per year)

○ Yes – irregularly (less than one pharmacist per year)

○ No

1.7. How do you rate the ration of regular customers to walk-in customers in your pharmacy? (Single-choice question)

○ More regular customers

○ More walk-in customers

○ Ratio is even

1.8. How many patients visit your pharmacy on an average day ? (Single-choice question)

○ Less than 70 patients

○ 70 – 250 patients

○ More than 250 patients

1.9. How many patients who were recently discharged from hospital (within the last week) come to your pharmacy with or without discharge prescriptions in an average month? (Single-choice question)

○ About one patient per month

○ 1 – < 5 patient(s) per week

○ 5 – 10 patients per week

○ > 10 patients per week

○ None

○ Unable to assess

1.10. From how many different hospitals were the patients who come to your pharmacy discharged? (Single-choice question)

○ One hospital

○ 2 – 4 hospitals

○ > 4 hospitals

○ Unable to assess

1.11. Which pharmaceutical services are currently provided by your pharmacy? (Single-choice question)

○ Extended medication consultation (pharmacy service)

○ Individual preparation of medication for patients (in personalized dispensers)

○ Structured care for patients in assisted living via contract

○ None of the before-mentioned services

1.12. Have you personally worked in a hospital pharmacy before 2017? (Single-choice question)

○ Yes

○ No

2. General questions about the new legal requirements (e.g. current barriers to implementation)

In the following part of the survey, the wording *“the new legal requirements”* refers to [1]:

- Handing out discharge summaries (at least as a preliminary version) at patients´ discharge from hospital
- Handing out medication lists (e.g. federal standard medication plan) for patients at their discharge
- After assessing the patients´ supply with required provision, if needed, discharge prescriptions are issued or medication dispensed

2.1. What changes in medication safety and continuous medication supply have been observed over the past five years since the new legal requirements came into force? (Single-choice question)

For each response item (2.1.a – 2.1.e) it should be chosen between: (i) has improved (ii) remained unchanged (iii) has deteriorated (iv) unable to assess.

- ○ Number of patients presenting medication lists from hospital
- ○ Patients´ knowledge about their current medication
- ○ Number of patients´ questions about their current medication
- ○ Number of patients to whose medication you as pharmacists have questions
- ○ Number of patients who are insufficiently supplied with required medicines

2.2. What drug-related problems do you typically observe in recently discharged patients? (Selection and wording of drug-related problems based on [2]) (Multiple-choice question)

- ○ Drug-drug interactions
- ○ Adverse drug events
- ○ Unnecessary duplications of medication (e.g. drugs with the same active ingredients and/or of the same therapeutic group)
- ○ Inappropriate dosing intervals (e.g. Administration of an antibiotic at 8am, 12am and 6pm instead of every 8 hours)
- ○ Inappropriate dosage regimes (e.g. in case of renal impairment)
- ○ Inappropriate treatment duration (e.g. short-term medication is prescribed as long-term treatment)
- ○ Inappropriate dose timing (e.g. taking diuretics in the evening)

- ○ Inappropriate drug selection (e.g. medication in discordance with guidelines)
- ○ Inappropriate application form (e.g. powder inhaler for patients with weakened breathing)
- ○ Difficulties in administering the medication (e.g. applicating inhaler or dividing tablets)
- ○ Contraindications (e.g. due to comorbidities, allergies or age)
- ○ Drug-food interactions (e.g. grape fruit)
- ○ Overuse (e.g. medication without indication)
- ○ Underuse (e.g. indication without medication)
- ○ Patients´ lack of knowledge about new or changed medication
- ○ Non-adherence
- ○ We do not observe drug-related problems
- ○ Unable to assess

2.3. For which patient group do you observe increased risks of drug-related problems during transitions of care? (Multiple-choice question)

- ○ Patients with polypharmacy (5 or more prescribed drugs)
- ○ Patients who take medications of certain therapeutic groups (e.g. anticoagulants, antibiotics, oral anticancer drugs)
- ○ Patients with limited mobility
- ○ Patients with communication difficulties (e.g. insufficient language skills or comprehension difficulties and failing linguistic ability)
- ○ Patients who are not supported by relatives or informal caregivers
- ○ Patients living assisted in nursing homes
- ○ Patients who are supported by formal outpatient care givers
- ○ There are no certain patient groups
- ○ Unable to assess
- ○ Others: (Text input option)

3. Medication information transfer – medication list

3.1. What proportion of recently discharged patients can present medication lists from hospital upon request? (Single-choice question)

- ○ More than half
- ○ About half
- ○ Less than half
- ○ None
- ○ Unable to assess

3.2. What do you use the medication lists in those cases for? (Multiple-choice question)

- ○ Patient counselling
- ○ Update and handing over medication lists to patients
- ○ Transferring the dosage regimes onto the drug packages
- ○ Documenting medication and its changes (e.g. in our own medical records)
- ○ Medication reviews
- ○ No further use
- ○ Unable to assess
- ○ Others: (Text input option)

3.3. What difficulties in using medication lists are currently observed in routine care? (Multiple-Choice question)

- ○ Insufficient comprehensibility for patients
- ○ Insufficient comprehensibility for pharmacists
- ○ Discrepancies in medication information between other documents (e.g. discharge summary)
- ○ Difficulties in timely accessibility of inpatient healthcare professionals in case of queries
- ○ No difficulties
- ○ Unable to assess
- ○ Others: (Text input option)

4. Continuous medication supply – discharge prescriptions

4.1. What proportion of recently discharged patients come to your pharmacy with discharge prescriptions? (Single-choice question)

- ○ More than half
- ○ About half
- ○ Less than half
- ○ We see only patients without discharge prescriptions.
- ○ Unable to assess

4.2. What proportion of those discharge prescriptions can be filled in and drugs dispensed in time? (Single-choice question)

- ○ More than half
- ○ About half
- ○ Less than half
- ○ None
- ○ Unable to assess

4.3. What difficulties are currently observed in working with discharge prescriptions in routine care? (Multiple-choice question)

- ○ Missing or inappropriate compliance to the obligation to be issued by senior physician
- ○ Missing or inappropriate compliance to the smallest packages size
- ○ Missing or inappropriate compliance to using the correct template
- ○ Missing or inappropriate compliance to using the institutional identification
- ○ Missing or inappropriate compliance to further requirements (e.g. documenting dosage regimes, signatures, …)
- ○ Patients come with expired prescriptions
- ○ Missing or ambiguous medication documentation on discharge prescriptions
- ○ Difficulties in timely accessibility of inpatient healthcare professionals in case of queries
- ○ Difficulties in timely drug dispensing as e.g. patients come into community pharmacies after their last order

- ○ Unavailability of prescribed active substances in the German market
- ○ Unavailability of prescribed package size in the German market
- ○ No difficulties
- ○ Unable to assess
- ○ Others: (Text input option)

4.4. What proportion of recently discharged patients lack the required medication? (Single-choice question)

- ○ More than half
- ○ About half
- ○ Less than half
- ○ None
- ○ Unable to assess

4.5. What difficulties are currently observed in handling discharge prescriptions of narcotics in routine care? (Multiple-choice question)

- ○ We do not receive discharge prescriptions for narcotics issued by hospitals
- ○ Missing or inappropriate compliance to the obligation to be issued by senior physician
- ○ Missing or inappropriate compliance to the smallest packages size
- ○ Missing or inappropriate compliance to further requirements (e.g. ambiguous prescription, missing dosage regimes, exceeding the quantity requirements, …)
- ○ Missing or ambiguous medication documentation on discharge prescriptions
- ○ Difficulties in timely accessibility of inpatient healthcare professionals in case of queries
- ○ Difficulties in timely drug dispensing as e.g. patients come into community pharmacies after their last order
- ○ Unavailability of prescribed active substances in the German drug market
- ○ Unavailability of prescribed package size in the German drug market
- ○ No difficulties
- ○ Unable to assess

- Others: (Text input option)

5. Medication information transfer – handling discharge summaries

5.1. Do patients bring discharge summaries to your pharmacy? (Single-choice question)

- Yes – about once a week
- Yes – less than once a week
- No

5.2. What do you use the discharge summaries for? (Multiple-choice question)

- Patient counselling
- Transferring the dosage regimes onto the drug packages
- Documenting medication and its changes (e.g. in our own medical records)
- Medication reviews
- No further use
- Unable to assess
- Others: (Text input option)

5.3. What changes in medication documentation in discharge summaries have been observed over the past five years since the new legal requirements came into force? (Single-choice question)

For each response item (6.3.a – 6.3.e) it should be chosen between: (i) has increased (ii) remained unchanged (iii) has decreased (v) unable to assess.

- Fulfilment of quality criteria of medication documentation (e.g. drug name, strength, dosage regimes, …)
- Correctness of medication documentation as in conformity with the medication list
- Comprehensibility for pharmacists
- Comprehensibility of documented changes in medication during hospital stay
- Comprehensibility for patients

6. Medication information transfer – patient consultation

6.1. About what type of medication do you talk about typically with recently discharged patients? (Multiple-Choice question)

- Newly prescribed medication
- Medication which was changed or ceased during hospital stay
- Drugs of certain therapeutic groups (e.g. anticoagulants, antibiotics, anticancer drugs)
- Drugs with special application forms (e.g. inhaler, pens, syringes)
- Medication which was handed over to patients at discharge
- No medication in particular or rather always different medication
- Unable to assess
- Others: (Text input option)

6.2. About what drug-related issues do you talk about typically in those “patient consultations” with patients and/or their relatives or informal caregivers? (Multiple-Choice question)

- Dosage regimes
- Preparation of medication to be administered (e.g. dividing tablets)
- Instructions how to administer special application forms (e.g. inhaler)
- Advices for administration (e.g. do not administer L-Thyroxin together with Calcium)
- Treatment duration
- Indication and potential benefits of the treatment
- Potential drug-drug interactions with further medication
- Potential adverse drug events
- Changes in medication which were made during hospital stay
- Storage of drugs at home
- Advices for drug monitoring (e.g. blood pressure)
- Unable to assess
- Others: (Text input option)

6.3. With which group of recently discharged patients do you observe in total more difficulties? (Single-choice question)

- Patients who come to our pharmacy directly after hospital discharge and bring discharge prescriptions

- ○ Patients who come to your pharmacy after they have visited the practice of their general practitioner after hospital discharge and thus, have e.g. new prescriptions and/or an updated medication list
- ○ There is no difference between those two groups
- ○ Unable to assess

7. Potential for improvement of discharge processes

7.1. How could you as community pharmacy better support patient after hospital discharge? (Text input option)

7.2. What would you need to do so? (Text input option)

References

[1] GKV-Spitzenverband, Kassenärztlichen Bundesvereinigung, Deutschen Krankenhausgesellschaft e. V. . Rahmenvertrag über ein Entlassmanagement beim Übergang in die Versorgung nach Krankenhausbehandlung nach § 39 Absatz 1a Satz 10 SGB V (Rahmenvertrag Entlassmanagement) in der Fassung der 10. Änderungsvereinbarung vom 22.05.2023. https://gkv-spitzenverband.de/krankenversicherung/ambulant_stationaere_versorgung/entlassmanagement/entlassmanagement.jsp. Accessed 24 Nov 2023.

[2] ABDA - Federal Union of German Associations of Pharmacists. Grundsatzpapier zur Medikationsanalyse und zum Medikationsmanagement https://www.abda.de/themen/arzneimitteltherapiesicherheit/foerderinitiative-pharmazeutische-betreuung/medikationsmanagement/. Accessed 29 Feb 2024.
