# Supplementary material for: Continuity of medication information transfer and continuous medication supply during hospital-to-home transitions - nationwide surveys in hospital and community pharmacies after implementing new legal requirements in Germany
Source: BMC Health Serv Res. 2024 Aug 27;24:993. doi: 10.1186/s12913-024-11208-4 (PMC11348592; doi:10.1186/s12913-024-11208-4)
Supplement: Supplementary file 2 — Supplementary Material 2: Additional File 2 presents the full response patterns to the questions. [file 12913_2024_11208_MOESM2_ESM.pdf]

Additional file 2

Continuity of medication information transfer and continuous medication supply at hospital-to-home transitions - nationwide surveys in hospital and community pharmacies after implementing new legal requirements in Germany

Sophia Klasing [1,2], Frank Dörje [2,3], Heike Hilgarth [2,4], Nadine Metzger [2,5], Ina Richling [2,6,7], Hanna M. Seidling [1,2] *

* Correspondence

Hanna M. Seidling

Hanna.seidling@med.uni-heidelberg.de

A1 – response rates of the pharmacies per chamber of pharmacist.

Chamber of pharmacist	Response rate per total number community pharmacies of the respective chamber of pharmacist	
	Hospital pharmacies [1]	Community pharmacies [2]
Baden-Wuerttemberg	37.0 %	3.3 %
Northrhine	45.9 %	5.7 %
Rhineland Palatinate	34.6 %	1.8 %
Saarland	42.9 %	7.4 %
Saxony	31.6 %	5.2 %
Saxony-Anhalt	28.6 %	8.9 %
Schleswig Holstein	20.0 %	6.1%
Thuringa	6.3 %	3.6 %
Westphalia-Lippe	23.8 %	5.5 %
Bavaria	27.8 %	5.2 %
Berlin	18.2 %	4.0 %
Brandenburg	23.1 %	5.9 %
Bremen	0 %	7.5 %
Hamburg	50.0 %	1.6 %
Hesse	40.0 %	5.4 %
Mecklenburg Western Pomerania	11.1 %	8.6 %
Lower Saxony	40.7 %	0.4 %

[1] ABDA - Bundesvereinigung Deutscher Apothekerverbände. Krankenhausapotheken 2023 (Landesapothekerkammern ABDA_Statistik: Stichtag 30.06.2023) (provided for naming).

[2] ABDA - Bundesvereinigung Deutscher Apothekerverbände. Entwicklung der Apothekenzahl 1. Halbjahr 2023 (Landesapothekerkammern ABDA_Statistik: Stichtag 30.06.2023) (provided for naming).

A2 – hospital pharmacies´ perceptions of the implementation of discharge summaries, medication lists, discharge prescriptions and dispensing medication in routine care. Questions were translated into English for the purpose of publishing.

CONTINUITY OF MEDICATION INFORMATION TRANSFER									
				CONTINUOUS MEDICATION SUPPLY					
What proportion of recently discharged patients can present **medication lists** upon request? (Single-choice; percentage of community pharmacies of N = 796)		Do recently discharged patients bring **discharge summaries** to community pharmacies? (Single-choice; percentage of community pharmacies of N = 796)		What proportion of recently discharged patients come to your pharmacy with **discharge prescriptions**? (Single-choice; percentage of community pharmacies of N = 796)		What proportion of those **discharge prescriptions can be filled in** and drugs dispensed in time? (Single-choice; percentage of community pharmacies of N = 611)		What proportion of recently discharged patients **lack the required medication**? (Single-choice; percentage of community pharmacies of N = 796)	
more than half	31.8 %	about once a week	34.0 %	more than half	27.6 %	more than half	73.3 %	more than half	12.3 %
about half	20.0 %	less than once a week	48.7 %	about half	21.6 %	about half	18.0 %	about half	18.7 %
less than half	29.0 %			less than half	27.5 %	less than half	7.0 %	less than half	47.7 %
none	4.4 %	no	17.2%	There were no patients with discharge prescriptions	0.4 %	There were no patients with discharge prescriptions	0.3 %	none	4.6 %
unable to assess	14.8 %			unable to assess	22.9 %	unable to assess	1.1 %	unable to assess	16.6 %
no answer	0.0 %	no answer	0.0 %	no answer	0.0 %	no answer	0.2 %	no answer	0.0 %

A3 – difficulties in routine care. Questions were translated into English for the purpose of publishing.

		**Medication list**		**Discharge summary**	**Discharge prescription**		**Dispensing medicines at discharge**
What difficulties are currently observed in routine care? (MC)		Percentage of participating hp (n = 18)	cp (n = 643)	hp (n = 11)	hp (n = 20)	cp (n = 611)	hp (n = 22)
… preparing/ issuing by inpatient health care professionals	assessment of actual patients´ need for medication supply				25.0 %		22.7 %
	timely availability of medication information	33.3 %		27.3 %	20.0 %		13.6 %
	missing or incomplete comparison with pre-admission medication and adaptions to the medication lists if needed	44.4 %		18.2 %			
	inpatient interprofessional and/or interdisciplinary communication	27.8 %		27.3 %	15.0 %		27.3 %
	insufficient information for patients						50.0 %
… resources	technical issues	50.0 %		0.0 %	55.0 %		
	shortage in time and personnel resources	77.8 %		63.6 %	60.0 %		27.3 %
	unavailability of prescribed active substances in the German drug market					65.1 %	
	unavailability of prescribed package size in the German drug market					60.7 %	

… compliance with formal requirements	using the correct template					24.5 %	
	prescribing the smallest packages				20.0 %	62.5 %	
	obligation to be issued by senior physicians				25.0 %	31.8 %	
	using the institutional identification					48.8 %	
	others (e.g. missing dosage)				35.0 %	75.1 %	
… use by outpatient health care professionals	comprehensibility for pharmacists		15.9 %				
	missing or ambiguous medication documentation					50.1 %	
	discrepancies in medication information between other documents (e.g. discharge summary)		49.8 %				
	timely accessibility of inpatient healthcare professionals in case of queries		73.4 %			79.2 %	
… patients	comprehensibility for patients		54.7 %				
	patients come with expired prescriptions.					68.7 %	
	Timely drug dispensing, e.g. patients come into community pharmacies after their last order.					60.7 %	
	recognizing their required medication in dispensed medicines						31.8 %
	no difficulties	0.0 %	7.9 %	9.1 %	0.0 %	0.9 %	9.1 %
	unable to assess	0.0 %	1.4 %	18.2 %	5.0 %	0.0 %	27.3 %
	no answer	0.0 %	0.3 %	0.0 %	0.0 %	0.0 %	9.1 %

Hp = hospital pharmacies; cp = community pharmacies; n = number

A4 – facilitators to implement the new legal requirements in inpatient routine care. Question was translated into English for the purpose of publishing.

What are the current facilitators to implement the new legal requirements in inpatient routine care? (MC)	Percentage participating hospital pharmacies (n = 111)
Software-based medication documentation processes	62.2 %
Successful interprofessional communication	36.0 %
Increase the number of pharmaceutical staff	16.2 %
Increase the number of further staff	9.9 %
Support of the hospital´s board of directors	15.3 %
No facilitators	14.4 %
Unable to assess	15.3 %
